# The migration ability of stem cells can explain the existence of cancer of unknown primary site. Rethinking metastasis

**DOI:** 10.18632/oncoscience.159

**Published:** 2015-05-01

**Authors:** Miguel López-Lázaro

**Affiliations:** ^1^ Department of Pharmacology, Faculty of Pharmacy, University of Seville, Spain

**Keywords:** Cancer of unknown origin, Cancer stem cells, Cancer therapy, Cells of origin in cancer, Stem cell model of cancer

## Abstract

Cancers of unknown primary site are metastatic cancers for which primary tumors are not found after detailed investigations. In many cases, the site of origin is not identified even on postmortem examination. These cancers are the fourth most common cause of cancer death. The biological events involved in the development of this type of cancers remain unknown. This manuscript discusses that, like metastatic cells, stem cells have a natural ability to migrate. A cancer of unknown primary site would form when deregulated, premalignant or cancerous stem cells migrated away from their natural tissue and gave rise to a cancer in a new site before or without generating a tumor in their original tissue. It is important to realize that forming a tumor in a tissue is not a prerequisite for stem cells to migrate away from that tissue. This view is in accordance with recent observations that strongly support the tumorigenesis model in which cancer arises from normal stem cells. Evidence has accumulated that cancer stem cells may play a key role in cancer progression and resistance to therapy. Successful treatment of cancer, including that of unknown primary site, may therefore require the development of therapies against cancer stem cells.

## INTRODUCTION

Metastasis is considered to be the process by which cancer cells leave a primary tumor and form one or several secondary tumors in other parts of the body; these secondary tumors are called metastatic tumors or metastases [1]. Histological analyses commonly show that cells from the primary and secondary tumors are similar and resemble those of the normal tissue of origin. In most cancer patients, the primary tumor is diagnosed before or at the same time than its metastases. Sometimes, metastatic tumors are found before standard diagnostic studies reveal the location of the primary site. But other times, metastatic tumors are found, and a primary tumor is not identified after detailed diagnostic investigations. These tumors generally contain poorly differentiated or undifferentiated cells, which makes the identification of the tissue of origin difficult. In some cases, the histology or location of the tumor strongly suggest a specific primary site; however, exhaustive investigations also fail to identify a primary tumor. These cancers are called cancers of unknown primary (CUP), cancers of unknown primary site, cancers of unknown origin, or occult primary cancers [2-4].

Cancer of unknown primary site is a heterogeneous group of cancers for which the anatomical site of origin remains occult after detailed investigations. In 15-25% of cases, the primary site is not identified even on postmortem examination. Cancer of unknown primary site accounts for approximately 3-5% of all human cancers. It is the seventh to eighth most frequent malignancy, and it is the fourth most common cause of cancer death in both sexes. In a third of patients diagnosed with CUP, three or more organs are involved at the time of diagnosis. The pattern of metastasis (frequency and location of metastases) in patients diagnosed with CUP may be significantly different from that in patients with known primary tumors. Most cancers of unknown primary site show an aggressive behavior and are incurable [2-4].

Since the site of the primary tumor usually dictates the treatment and expected outcome, the inability to identify a primary site raises concern among oncologists and patients. Current research and clinical efforts are made to develop and use knowledge and technology to locate the primary tumor or, at least, to reliably predict the tissue of origin. Primary and metastatic tumors commonly have similar expression profiles and molecular signatures. This makes immunohistochemical testing and tissue-of-origin molecular profiling useful tools for predicting the tissue of origin [3]. Recently, Polak *et al.* compared mutation densities to epigenetic profiles of normal and cancer cells from different tissues, and reported findings suggesting that the tissue of origin of a cancer may be accurately predicted based on the distribution of mutations along its genome [5]. Predicting the tissue of origin is particularly important for the types of CUP that respond relatively well to specific therapies (approximately 20% of CUP). When these cancers are ruled out, it usually becomes less important to find or predict the primary site. For example, in an analysis of several post-mortem cohort studies, the potential primary tumor was identified in 73% of patients, and the most common primaries were lung (27%) and pancreatic (24%) tumors [6]. The five-year relative survival rate for patients with lung cancer and pancreatic cancer with distant metastases is 4% and 2%, respectively [7]. Therefore, the identification of a primary site in patients with these two types of cancer would not have changed much their outcome.

Understanding the existence of cancers of unknown primary site and the mechanisms involved in their formation may lead to the development of better treatments. These treatments may be useful not only for patients with these cancers, but also for patients with other metastatic cancers. Currently, it is generally accepted that CUP exists because of our inability to identify the primary tumor due to clinical or technological inefficiencies, or because the primary tumor regresses or stays dormant after spreading the cancer cells that generate the metastases [2-4]. Some studies have shown molecular features shared by cancers of unknown primary origin. For example, a recent analysis of 1806 cases of cancer of unknown primary site has revealed that *TP53* is the most commonly mutated gene in these cancers [8]. However, the biological events that allow the primary site to remain occult after the development of metastases remain unknown [2-4].

### Stem cells have a natural ability to migrate

Recent evidence suggests that the biological events occurring during the development of metastasis are rather similar to those occurring during embryonic development. During embryogenesis, stem cells can invade tissues, move through the interior of the embryo, travel long distances, and establish in new places to participate in the formation of organs and tissues [9-11]. During metastasis, cancer cells can invade tissues, move through the lymphatic and circulatory systems, travel long distances, and establish in new tissues to form tumors [1,12]. The activation of the epithelial-mesenchymal transition (EMT) and the mesenchymal-epithelial transition (MET) seems to play a critical role in the migration ability of both embryonic stem cells and metastatic cells. The activation of these transition programs involves profound changes in cell morphology and behavior, including changes in cytoskeleton structure, cell polarity, cell-cell contact and extracellular matrix degradation. Recent data suggest that EMT activation is an early event in carcinogenesis, and that there is a crosstalk among EMT activation, the acquisition of molecular and functional traits of cancer stem cells, and the inactivation or mutation of p53 [9,10,13-15].

The migration ability of stem cells is repressed after embryonic development, but probably reappears during pathological conditions. Adult stem cells (also known as tissue stem cells) can increase their migratory activity when their microenvironment is altered [16]. These cells play a key role in tissue damage repair; tissue injury activates developmental programs that activate adult stem cell migration to the site of damage [17]. Interestingly, tissue injury may increase cancer risk [18-21]. Adult stem cells may also increase their migratory potential after accumulating specific DNA alterations. These premalignant stem cells may also acquire additional DNA alterations and become cancer stem cells (CSCs). These cancer cells seem to play a key role in tumor metastasis [12,22]. It has been proposed that CSCs can be stationary (which establish tumor growth) or mobile (which lead to tumor metastasis) [23], and these two populations of CSCs have been found in human cancer tissues [24].

**Figure 1:**
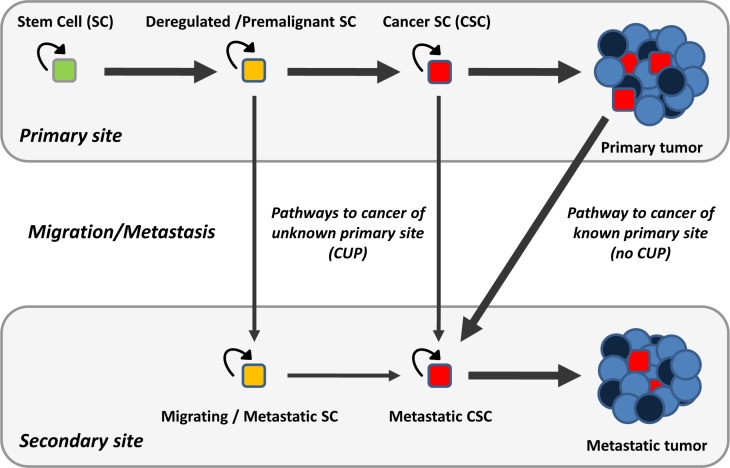
The migration ability of stem cells can explain the existence of cancer of unknown primary site A cancer of unknown primary site would form when deregulated stem cells, premalignant stem cells or cancer stem cells migrated away from their natural tissue (primary site) and formed a tumor in another tissue (secondary site) before or without generating a tumor in their primary location. Thick arrows represent the probably most common pathway for metastasis. Curved arrows represent self-renewal capacity. See text for further details.

The migration ability of stem cells can explain the existence of cancers of unknown primary site. A cancer of unknown primary site would form when deregulated, premalignant or cancerous stem cells migrated away from their natural tissue and gave rise to a cancer in the new site before or without generating a tumor in their original tissue. It is important to realize that forming a tumor in a tissue is not a prerequisite for stem cells to move away from that tissue; this is probably the key to understand the existence of cancer of unknown primary site. Stem cells can migrate from their natural tissue and initiate a cancer in the new site before generating a detectable tumor in their natural tissue. In this case, the primary tumor could be identified after some time. However, stem cells can also migrate away from their natural tissue without generating a cancer there. In this case, the “primary tumor” would never exist. This can explain why the primary site is not identified even on postmortem examination in many patients with these cancers (Figure 1).

### Cancer may arise from normal stem cells

This explanation for the existence of cancers of unknown primary site implies that these cancers arise either from normal stem cells or from non-stem cells that have activated stem cell programs. Here I discuss evidence suggesting that cancer, including that of unknown primary, may originate in normal stem cells.

Numerous experimental investigations have been conducted to identify the cells of origin in cancer [25]. These investigations both challenge and support the idea of cancer arising from stem cells [25-27]. For example, experimental data suggest that progenitor cells can dedifferentiate and develop stem-like properties; this has led to the proposal that cancer may start in progenitor cells [26]. Experimental data have also shown that long-term passaged stem cells become cancer cells in culture and generate solid tumors when injected into rodents [28]. It is important to note that all these investigations involve experimental manipulation. As discussed elsewhere [28], stem cells are defined in terms of their functional properties: long-term self-renewal capacity and differentiation potential. This presents an inherent problem because these properties can only be assessed by experimental manipulation, and experimental manipulation probably alters the functional properties of these cells. Just culturing stem cells *in vitro* may induce profound changes in their proliferative rates, cellular fate or behavior. *In vivo*, stem cells are established in niches. Stem cell niches are microenvironments that protect the stem cells and regulate the way they behave. The stem cell niche provides support and signals that regulate their proliferative rates, self-renewal, differentiation and migration activities [16]. Stem cells cultured *in vitro* are deprived of their niches, which may completely change their biological behavior. This may explain, for example, that stem cells that are resting *in vivo* may become proliferative in cell culture. In addition, the studies aimed at identifying the cells of origin in cancer usually involve a genetic or chemical manipulation of the cells [25]. We should therefore interpret the findings of these experimental studies cautiously.

Solid biological concepts may provide more reliable information on the cells of origin in cancer. The long lifespan and self-renewal capacity of stem cells support the idea of cancer arising from these cells. These biological properties indicate that stem cells have the opportunity to accumulate sufficient DNA alterations to generate cancer cells [29]. Three reasons are commonly used to challenge this idea. First, cancer probably starts in cells with high proliferative rates, and the adult stem cells of some tissues do not divide often. Second, stem cells represent a small population within a tissue, and the probability of a stochastic carcinogenic hit is lower in a small population than in a large population. Third, carcinogenesis depends on the acquisition of advantageous cell phenotypes, and these phenotypes are less likely to occur when a cell self-renews than when a cell differentiates [26]. Recognizing that life starts before birth makes the first two challenging reasons difficult to accept. First, stem cells divide very actively during embryonic development.

During this relatively short period, one cell (zygote) gives rise to the billions of cells that form a developed embryo [30]. The genome of the adult stem cells at the time of birth has already been copied many times, and probably contains many heritable alterations in relation to that of the zygote. In some adult stem cells, these alterations may contribute to a possible carcinogenesis process occurring later in life. They may also increase their proliferative rates during adulthood. Second, stem cells are the only cell population at the initial stages of embryonic development. Stem cells are therefore the only cells exposed to the acquisition of carcinogenic damage during this period of intense mitotic activity. Third, the chance of a carcinogenic hit during cell division is probably similar in a cell that self-renews to produce two undifferentiated daughter cells than in a cell that gives rise to two differentiated daughter cells. The limited fidelity of DNA polymerases is known to be an important source of DNA mutations during cell division. DNA polymerases probably insert similar levels of incorrect nucleotides in the DNA of the daughter cells when copying the DNA of a stem cell that self-renews than when copying the DNA of a progenitor cell that differentiates. The acquisition of advantageous cell phenotypes is probably relevant at late stages of carcinogenesis; however, this factor is unlikely to determine what type of cell is more prone to acquire the first carcinogenic hit. Overall, the idea of cancer arising from normal stem cells has a solid biological basis.

**Figure 2:**
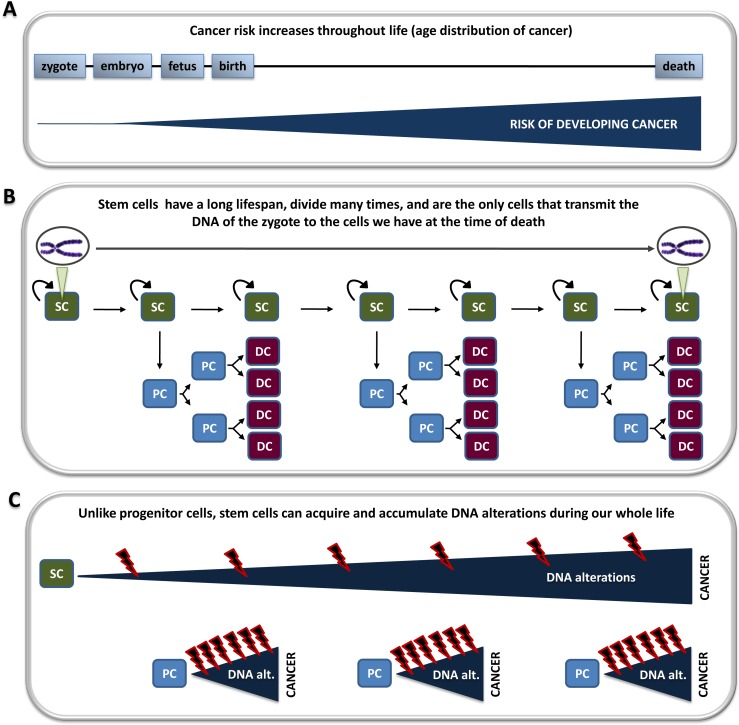
The stem cell model of cancer can explain the age distribution of cancer A) Age is the most important cancer risk factor, and cancer incidence increases with age. B) Stem cells self-renew, have a long lifespan, and are the only cells that transmit the DNA of the zygote to the cells we have at the time of death. Stem cells can differentiate into progenitor cells and eventually into differentiated cells. C) Stem cells can acquire and accumulate DNA alterations during our whole life. Because progenitor cells lack an innate ability to self-renew and have a relatively short lifespan (until they fully differentiate), they can acquire and accumulate DNA alterations only during relatively short periods of our life. The model in which cancer arises from stem cells fits the age distribution of cancer better than the model in which cancer initiates in progenitor cells. Curved arrows represent self-renewal capacity. SC: stem cell; PC: progenitor cell; DC: differentiated cell.

It is important to recognize that the first carcinogenic hit does not necessarily have to be the first mutation occurring in one of the genes whose involvement in cancer development is understood at a given moment. The first carcinogenic hit should be referred to as the first heritable change (e.g., DNA mutation, epigenetic change or chromosome alteration) that participates in the formation of a cancer. The first hit may be a DNA alteration with biological implications not yet understood, and may occur in a non-coding DNA sequence [31].

Observational studies do not involve experimental manipulation and may provide reliable information regarding the cells of origin in cancer. Numerous observational studies have shown that age is the most important cancer risk factor [7,32-35]. For example, recent data show that approximately 4.4% of people under 50 years old are diagnosed with cancer in the US; this percentage increases to 6.3% in people in their 50s, to 12.5% in people in their 60s, and to 31.2% in people older than 70 years old [7]. These studies indicate that our cancer risk increases during our whole life. Stem cells are the only cells that transmit the genome of the zygote to the cells we have at the time of death and, therefore, are the only cells that can acquire and accumulate DNA alterations during our whole life. Progenitor cells lack an innate ability to self-renew and have a relatively short lifespan. Progenitor cells, therefore, can acquire and accumulate DNA alterations only during relatively short periods of our life. If cancer arose from progenitor cells, a progenitor cell produced when we are 10 would have the same probably to initiate a cancer than a progenitor cell produced when we are 30 or 50. After a lag period of e.g. 20 years, our risk of having cancer would be similar in our 30s, 50s or 70s. Cancer statistics show otherwise. The stem cell model of cancer fits the age distribution of cancer better than the progenitor cell model of cancer (Figure 2). Cancer statistics also show that cancer incidence decelerates late in life, i.e., cancer incidence rises with age, but the rise occurs more slowly in later years. Interestingly, an overall decline in tissue regenerative potential also occurs in later years, which is attributed to a decline in stem cell functionality with age [36].

The observation that some tissues give rise to cancers millions of times more often than other tissues has long puzzled cancer researchers. This observation cannot be explained by different levels of exposure to carcinogens. For example, melanocytes and basal epidermal cells of the skin are exposed to similar levels of the same carcinogen (ultraviolet radiation); however, basal cell carcinomas are much more common than melanomas [37]. C. Tomasetti and B. Vogelstein [37] have recently provided an explanation for this observation. They found that there exists a highly positive correlation (Spearman's rho = 0.81; P < 3.5 × 10^−8^) between the number of normal stem cell divisions in a tissue and the lifetime risk for developing cancer in that tissue. This striking correlation applied to 31 cancer types and extended across five orders of magnitude. No environmental or inherited factor is known to correlate so strongly with cancer incidence across tissues [37]. In simple terms, the data provided by C. Tomasetti and B. Vogelstein indicate that if the normal stem cells from one of our tissues divide once, our cancer risk in that tissue is approximately 1X. If they divide 10 times, our cancer risk is 10X. If they divide 1,000 times, our cancer risk is 1,000X. And if the normal stem cells from one of our tissues divide 100,000 times, our cancer risk in that tissue is approximately 100,000X. This strongly suggests that the main reason we have cancer is that our normal stem cells divide. A reasonable way to interpret this is that cancer arises from normal stem cells. The other option would be difficult to accept. If cancer arose and developed in non-stem cells, we would have to admit that stem cell division is the main determinant for the neoplastic transformation of non-stem cells. This would mean that any time a particular cell divides, another cell is transformed. It makes much more sense to think that normal stem cells acquire and accumulate carcinogenic damage when they divide than to think that their division inflicts carcinogenic damage on non-stem cells.

## CONCLUDING REMARKS

Currently, metastasis is considered to follow the next sequence: invasion and intravasation of cancer cells from the primary tumor, dissemination through the circulation, extravasation in different organs, survival on arrival, settlement into latency, reactivation, and overt colonization with generation of a new macroscopic tumor [1,12]. If metastasis is the process by which cancer cells leave a primary tumor and form secondary tumors in other locations [1,12], the existence of cancers of unknown primary site can be seen as a biological mystery [4]. However, if we challenge this prevailing view and consider that the cells that leave the primary tissue do not necessarily have to be cancer cells from a tumor, the existence of cancers of unknown primary site becomes a plain biological process. The injection of morphologically normal breast cells from genetically engineered mice into the tail veins of other female mice resulted, after oncogene induction, in the formation of tumors in the lungs but not in the breast [38]. Although these cells were genetically and chemically manipulated, these experiments show that the formation of a primary tumor is not a requisite for the formation of metastatic tumors. Metastasis should be seen as the process by which cells from a tissue form tumors in other tissues. All cancers, including those of unknown primary site, fit in this simple definition (Figure 1).

The term occult primary tumor should be avoided in the absence of a primary tumor, because it implies that the tumor exists and is hidden. This term may cause anxiety among clinicians and patients, who may think that their evaluation has been deficient. In many cancers of unknown primary site, primary tumors are never found. In an analysis of post-mortem cohort studies, primary tumors were not identified in 27% of patients [6]. In some cancers of unknown primary site, the histology or location of the tumor strongly suggest a specific primary site. For example, the identification of adenocarcinomas in isolated unilateral axillary lymph nodes in women with CUP strongly suggests that these tumors originate in the breast. Breast tumors commonly metastasize to this site, and this type of CUP is found almost exclusively in women. However, breast tumors were not identified in 28% of 446 women who were diagnosed with this type of CUP and underwent mastectomy and subsequent histological analysis [2,39]. The terms primary tumor and secondary tumor should also be avoided in the absence of a primary tumor. In CUP patients, the terms primary site and tumor of unknown primary site should be used instead, at least until a possible primary tumor is eventually found.

Not all cells can leave their natural tissue and form tumors in other locations. The cell needs migration ability to leave its tissue and reach the new one, proliferative capacity to accumulate enough DNA alterations to produce a cancer cell, and self-renewal capacity so that its only fate is not the generation of non-dividing differentiated cells. Stem cells constitute the only cell population with these three biological properties under physiological conditions. This implies that cancers of unknown primary site originate in stem cells, or in non-stem cells that have acquired these biological properties.

Non-stem cells (e.g., progenitor cells) can acquire DNA alterations. In some cases, these alterations may result in the activation of stem-cell programs, and may even determine whether or not we will eventually have a cancer. But this does not imply that these cancers arise from non-stem cells, because the stem cells from which they derive may have DNA alterations that also participate in the formation of these cancers. Three lines of evidence discussed in this manuscript suggest that the majority of human cancers may arise from normal stem cells. First, cell division is a major source of DNA alterations, stem cells constitute the only cell population during the initial period of life (early stages of embryonic development), and stem cells divide very actively during this period. This means that it is likely that stem cells acquire DNA alterations during embryonic development, which may play a role in a possible carcinogenic process occurring later in life. Because the first carcinogenic hit may be a DNA alteration with biological implications not yet understood, we cannot exclude the possibility that an important percentage of cancers may start before birth. Second, stem cells are the only cells that transmit our DNA during our whole life, and the risk of developing cancer is known to increase during our whole life. The observation that cancer risk increases with age can be better explained by a model in which cancer initiates in stem cells than by a model in which cancer initiates in progenitor cells (Figure 2). Third, if cancer initiated and developed in non-stem cells, the presence or biological activity of stem cells in a tissue would not significantly modify the risk of developing cancer in that tissue. However, recent observations strongly suggest that the number of normal stem cell divisions in a particular tissue seems to be the main determinant for developing cancer in that tissue [37].

If cancer arises from normal stem cells, these cells would eventually become cells with the ability to generate tumors, i.e., cancer stem cells (CSCs). In other words, a CSC would be a stem cell that has developed a long-term ability to generate heterogeneous tumor populations. This view of CSCs considers their origin (normal stem cells), their self-renewal capacity (ability to copy themselves during long periods), and their differentiation potential (ability to generate heterogeneous cellular populations). These cells would develop in all types of cancer, including those in which they have not yet been identified. Some CSCs would self-renew, and others would differentiate and give rise to cells without self-renewal ability (progenitor cancer cells) and without self-renewal ability and proliferative capacity (differentiated cancer cells). All these cancer cells would contribute to the cellular heterogeneity of tumors [40]. A CSC located in a tissue different from that of the normal stem cell from which it originates would be a metastatic cancer stem cell. This view implies that the stem cell can migrate to the new tissue before or after becoming a CSC. It is important to realize that the process by which a normal cell becomes a cancer cell does not necessarily have to start and complete in the same tissue.

CSCs seem to play a crucial role in tumor growth, metastasis and resistance to therapy [22,41]. Successful cancer therapy may therefore require the elimination of these cells. Experimental data indicate that non-stem cancer cells may dedifferentiate into CSCs [27,42-45]. This means that, if we only eliminate CSCs, non-stem cancer cells may generate new CSCs and repopulate the tumor [42]. Successful therapy may therefore require the elimination of CSCs and their progeny. Clinical trials are ongoing to test the efficacy of drugs targeting CSCs, alone and in combination with conventional drugs targeting non-stem cancer cells [46].

Anti-CSC therapies might show efficacy in a wide range of CUP patients. Histological analyses of many tumors from CUP patients show populations of poorly differentiated or undifferentiated cells. Since stem cells constitute the less differentiated cell type, the undifferentiated cells of these tumors might be CSCs; these cells would play a role in the known aggressive behavior and therapeutic resistance of these cancers. In addition, the prognosis for most CUP patients is poor even when the primary site is predicted. The identification of a possible primary site allows CUP patients to be treated with the therapies used in metastatic patients with known primary tumors. These therapies, however, are generally not curative. CUP patients might therefore benefit from participating in clinical trials evaluating new anti-CSC treatments.

The lack of effective treatments for most CUP patients should not justify their inclusion in clinical studies testing experimental therapies not properly validated in preclinical models. Clinical trials in oncology have the highest failure rate compared with other therapeutic areas [47]. Most of the drugs that show remarkable anticancer effects in preclinical models fail when tested in cancer patients. Part of these failures may be due to poor preclinical designs, in which the needs of cancer patients are often misunderstood [48]. Cancer patients do not need new drugs that target their cancer cells at low concentrations if they also target their normal cells at similar concentrations. Cancer patients will probably not benefit from drugs that induce marked tumor shrinkages in animal models that do not represent their disease. Cancer patients need drugs that improve the efficacy of the existing treatments. Selectivity (*in vitro*) and survival rate (*in vivo*) are probably the most reliable parameters to predict drug efficacy in cancer patients [48]. *In vitro*, the new treatment should improve the selectivity of the existing drugs when tested in cancer cells versus a variety of normal cells (non-stem cells and stem cells from a variety of healthy tissues) [48-50]. The efficacy of a new drug combination should be assessed by testing in cancer cells *versus* non-malignant cells if it improves the selectivity of the standard treatment, and not by testing in cancer cells if its cytotoxicity is enhanced (potentiation factor) or synergistically increased (combination index) in relation to the cytotoxicity induced by each drug individually [48]. *In vivo,* the new treatment should improve the survival rate of the existing therapy in animal models representative of the patients who would eventually receive the new drugs [48]. Selecting animal models of metastasis is essential if the new drugs are intended for CUP patients or for other patients with metastatic disease. It should be noted that CSCs may constitute a minority population within a tumor (perhaps 1 to 3% [46]), and that a remarkable tumor shrinkage may leave the whole CSC population unaffected.

In summary, evidence suggests that the majority of human cancers may originate in normal stem cells. Cancers of unknown primary site probably exist because stem cells (deregulated, premalignant or cancerous) migrate away from their natural tissues and generate tumors in other locations before or without generating tumors in their natural tissues. Metastasis should be considered as the process by which cells from a tissue give rise to tumors in other locations.
